# Neural Decoding Using a Parallel Sequential Monte Carlo Method on Point Processes with Ensemble Effect

**DOI:** 10.1155/2014/685492

**Published:** 2014-05-18

**Authors:** Kai Xu, Yiwen Wang, Fang Wang, Yuxi Liao, Qiaosheng Zhang, Hongbao Li, Xiaoxiang Zheng

**Affiliations:** ^1^Qiushi Academy for Advanced Studies, Zhejiang University, Hangzhou 310027, China; ^2^Department of Biomedical Engineering, Zhejiang University, Hangzhou 310027, China; ^3^Key Laboratory of Biomedical Engineering of Ministry of Education, Zhejiang University, Hangzhou 310027, China

## Abstract

Sequential Monte Carlo estimation on point processes has been successfully applied to predict the movement from neural activity. However, there exist some issues along with this method such as the simplified tuning model and the high computational complexity, which may degenerate the decoding performance of motor brain machine interfaces. In this paper, we adopt a general tuning model which takes recent ensemble activity into account. The goodness-of-fit analysis demonstrates that the proposed model can predict the neuronal response more accurately than the one only depending on kinematics. A new sequential Monte Carlo algorithm based on the proposed model is constructed. The algorithm can significantly reduce the root mean square error of decoding results, which decreases 23.6% in position estimation. In addition, we accelerate the decoding speed by implementing the proposed algorithm in a massive parallel manner on GPU. The results demonstrate that the spike trains can be decoded as point process in real time even with 8000 particles or 300 neurons, which is over 10 times faster than the serial implementation. The main contribution of our work is to enable the sequential Monte Carlo algorithm with point process observation to output the movement estimation much faster and more accurately.

## 1. Introduction

Brain machine interfaces (BMIs) attempt to build direct links between brains and artificial devices, such as computer cursors and robotic arms [1–5]. They are considered as potential solutions to help paralyzed patients restore motor control, especially for those suffering from stroke, spinal cord injury, or amyotrophic lateral sclerosis [6–8]. In the past decade, the research on BMIs has made a great progress due to the rapid growth and development in neuroscience, computer science, and engineering. Many experimental demonstrations have shown the ability to estimate continuous movement of the limbs by exploiting the spatial and temporal structure of the motor cortical activity [9–11].

To make the direct control of the prosthetic devices practical for those patients, the estimation of the movement should be highly accurate and fast enough for real-time implementation. Several signal processing approaches have been applied to extract the functional relationship between the neural activity and the corresponding movement [12–18]. Recently, sequential Monte Carlo estimation with point process observation (SMCPP) is proposed to decode the spike trains, in which the spike trains are regarded as point processes and the spiking timing information is exploited by estimating the instantaneous firing rate in a much shorter interval (~10 msec) [19–21], while this information is discarded by many previous decoding algorithms which directly predict the movement from binned spike trains [12, 13, 18]. In addition, compared with many state-space model based algorithms, such as Kalman filter and point process adaptive filter [15, 16, 22, 23], there is no restriction on the posterior distribution of the state, which makes the SMCPP more flexible and suitable for the highly nonlinear systems such as BMIs. The experiment has demonstrated that the removal of the Gaussian assumption on state distribution and the utilization of the neural tuning model to estimate firing rate could increase the decoding accuracy [20].

Although the SMCPP performs well in previous studies, several issues can be further improved. The first one is the tuning model, which reflects the physiological knowledge of neurons responding to stimuli. Properly building the tuning model helps the SMCPP estimation since the posterior state density is updated based on the discrepancy between the actual neural firings and the instantaneous firing rates estimated from the model. In previous studies, the tuning models in SMCPP are usually either a parametric or nonparametric function that assume firing rates of neurons are only dependent on the kinematics. However, recent studies have demonstrated that they also depend on extrinsic covariates, as well as many other factors. Truccolo et al. find that the spiking of a single neuron could be better predicted by the spiking history of ensemble [24, 25]. Pillow et al. analyze the correlated firing in a population of macaque parasol retinal ganglion cells and find that the spike times can be more accurately predicted when the spiking of other neurons is taken into account. Furthermore, the optimal, model-based decoding can extract 20% more information when the ensemble activity is included in the tuning function [26]. However, in the above study, the stimulus is binary sequence and the spikes are recorded from retinal cells. Another way to build a better tuning model is to explain the unknown factors using multidimensional hidden states [27, 28]. Although the results demonstrated that this approach can achieve a better decoding accuracy, a potential problem is that the hidden state needs to be estimated by an Expectation Maximization algorithm at every iteration, which may result into instability and extra computational complexity. Another issue of the SMCPP is the high computational complexity because of the posterior density estimation by large number of particles, which brings the challenge to the real-time BMI systems. To increase the decoding speed, the main method before is to increase the processor clock speed, while it becomes plateaued in recent years. An alternative way is to add multiple cores into CPU. However, the number of cores in most commercially available CPUs is 2 or 4 and some may be up to 8. Another solution is to implement the decoding algorithm on dedicated hardware architectures such as FPGA [29–31]. But the hardware-based solution is often difficult to develop and maintain and lacks flexibility.

In this paper, we attempt to address the issues of the SMCPP mentioned above, making the decoding algorithm more efficient and applicable for the high-performance BMI systems. Firstly, we propose to extend the state-space model [20] by incorporating the recent ensemble activity into the tuning functions of neurons and the state transition model. The predicting power of tuning models with and without ensemble activity is evaluated and compared. The continuous kinematics of a monkey performing a target-pursuit task are decoding from the neural recordings in the primary motor cortex using the SMCPP based on such new models. The statistical performances are evaluated on multiple days. We do the experiment to see whether the decoding accuracy of the SMCPP can be improved when the ensemble effect is taken into account. Another contribution of our work is the speedup of the decoding algorithm for the demands of real-time implementation. We implement the SMCPP in a fully parallel way based on CUDA, which is a technology that can increase the computing performance dramatically by utilizing the hundreds of cores in graphics processing unit (GPU). Significant improvement has been obtained by using the graphics processing unit to perform the feature extraction in real-time brain computer interface [32]. In our implementation, the particles are propagated and updated in parallel. We use the parallel prefix scan algorithm to perform the weight summation which is often done serially before. The decoding speed of the proposed algorithm is observed to examine whether it satisfies the requirement of real-time BMI systems especially when the number of particles is large. Due to the great development of the neural recording technology in the last decade, hundreds of neurons can be simultaneously recorded to build a high-dimensional data space [33]. We apply our proposed algorithm on such a scenario to validate its real advantages.

This paper is organized as follows.  Section 2 introduces data recording and the BMI task. The details of SMCPP algorithm, the corresponding neural tuning model, the state transition model, and the parallel implementation are described in Section 3. In Section 4, we evaluate the predicting power of different tuning models and compare the decoding accuracy using sequential Monte Carlo algorithm with and without including the recent ensemble activity. In addition, we observe the decoding speed of our proposed algorithm to see whether it fulfills the requirement of real-time BMI systems. We discuss the results and conclude in Section 5.

## 2. Experiment Setup and Data Recording

The paradigm of the motor brain machine interface was designed and implemented in the Qiushi Academy for Advanced Studies at Zhejiang University. An adult male monkey was trained to perform a two-dimensional target-reaching task. After a target circle was presented on a computer screen, the monkey moved the cursor towards the target by controlling a handheld joystick. When the target was intersected with the cursor for a certain time, it would disappear and another target would show in a new position nearby the current one. The monkey was rewarded when the task was performed successfully for a while. The corresponding position of the joystick was recorded continuously with a sampling rate of 20 Hz, the velocity was estimated as the difference between current and previous positions, and the acceleration was estimated as the first-order difference from the velocity.

**Figure 1:**
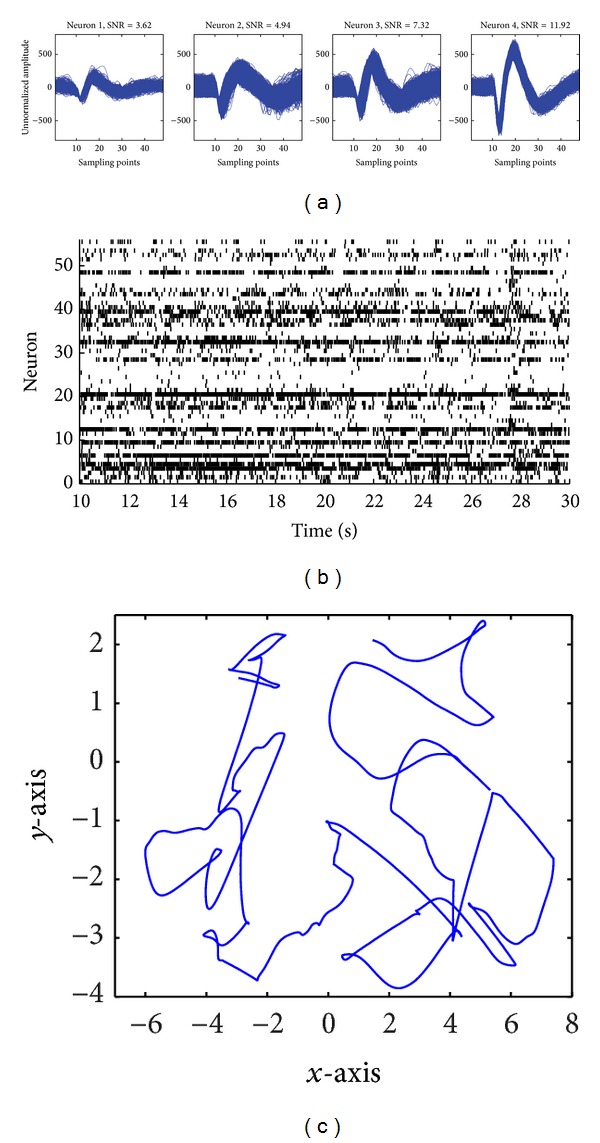
(a) The spike waveforms from 4 neurons with different SNRs. (b) The raster graph of the population activities during the task. (c) The arm trajectory in the 2D plane corresponding to the ensemble activity in (b).

A Utah array (1.0 mm electrode length, 96 channels with ICS-96 connector, Blackrock Microsystems) was chronically implanted in the arm area of primary motor cortex (M1) contralateral to the hand performing the task. Details about the surgical procedure could be found in [34, 35]. After the surgical procedure, a 128 Cerebus Data Acquisition system (Blackrock Microsystems, USA) was used to record the neuronal action potentials during the experiment. The sampling rate was 30 kHz and the action potentials were detected by a thresholding method. The waveforms of the spikes were amplified and band-pass filtered from 250 Hz to 7.5 kHz. Only the neurons whose firing rates were larger than 1 Hz and SNR that were larger than 2 were isolated to avoid the nonrobustness during the computation.  Figure 1(a) depicts the spikes recorded from 4 neurons with different SNRs.

The time of each spike was recorded and the firing rate was computed as the number of spikes within 10 ms time windows. We found that over 99% of these counts were either 0 or 1, which made the ensemble activity as multichannel point process observation. Meanwhile, all the kinematics recorded were interpolated and synchronized with the spike trains.  Figure 1(b) shows a raster graph of the ensemble activity during a time interval, and Figure 1(c) plots the corresponding trajectory of the position in 2D plane.

## 3. Methods

### 3.1. Tuning Function with Ensemble Correlation

Given a time interval (0 *T*], a spike train consists of *J* spikes of a neuron observed at time 0 < *u*_1_ < *u*_2_ < ⋯<*u*_*J*_ < *T*. Therefore, it can be regarded as a point process which is composed of a set of binary events that occur in continuous time, which can be fully characterized by its conditional intensity function:

(1) $$\begin{array}{c} \lambda \left(t\right)=\underset{\Delta \to 0}{\lim}\frac{P\left(N\left(t+\Delta \right)-N\left(t\right)=1\right)}{\Delta }, \end{array}$$

where *N*(*t*) is the number of spikes fired in the time interval (0 *t*]. Based on the theory of point process, if Δ is small enough, the probability of observing a spike in the interval between *t* and *t* + Δ can be well approximated by the following equation:

(2) $$\begin{array}{c} P\left(dN\left(t\right)\right)=\exp\left(dN\left(t\right)\log\left(\lambda \left(t\right)\Delta \right)-\lambda \left(t\right)\Delta \right), \end{array}$$

where *dN*(*t*) is the activity of the neuron at time *t*. If there is a spike at time *t*, then *dN*(*t*) equals 1, otherwise 0.

The conditional intensity functions of spike trains can be defined as the neuronal tuning functions which reflect the tuning property of neurons and characterize the relationship between the covariates and the neuronal response. The function should be designed properly because in decoding stage the posterior state distribution is updated based on the discrepancy between the firing of neurons and the instantaneous firing rate *λ*(*t*) estimated from it. In previous decoding algorithms, the tuning function was usually assumed to be only dependent on the current kinematics *x*(*t*). However, the studies in neuroscience demonstrate that the spiking activity of a single neuron is dependent not only on external covariates, but also on the spiking history of the neuron itself and spiking activities of other neurons due to the coupling between them. We propose to apply a more general tuning function which extends the traditional ones and estimate the instantaneous firing rate based on both the kinematics and recent ensemble activity. Let *x*_*t*_ and *H*_*t*_ represent the kinematics and the recent ensemble activity at time *t*, respectively. More specifically, *H*_*t*_^*i*^ is defined as the number of spikes fired during the time interval [*t* − *l* *t*) for the neuron *i*, where *l* is the time length which will be determined in the following analysis. Then our tuning function can be formulated as

(3) $$\begin{array}{c} \lambda \left(t\right)=\lambda \left(x_{t},H_{t}\right)=\exp\left(\alpha_{0}+\overset{D}{\underset{i=1}{\sum }}\alpha_{i}x_{t}^{i}+\overset{C}{\underset{j=1}{\sum }}\beta_{j}H_{t}^{j}\right), \end{array}$$

where *D* is the dimension of the kinematic vector, exp⁡(*α*_0_) is the background firing rate, *α*_*i*_ is the modulation in firing rate of the *i*th component of kinematic vector, *C* is the number of neurons in the ensemble, and *β*_*j*_ represents the influence of the *j*th neuron on the target neuron. The above equation takes the movement, the spiking history of the target neuron, and the contributions of other neurons into account and is referred to as full tuning model in the following. By contrast, the tuning equation which only depends on the movement is referred to as mov tuning model. It is similar to (3) but without the third term in the exponential function.

In the full tuning model, the time length *l* of the recent ensemble activity is a parameter that needs to be determined. If the value of *l* is too small, then information contained in the rest of ensemble activity will be wasted. While if the value is too large, which means too much ensemble activity is included, it is likely to incorporate irrelevant information which could bias the estimation of the firing rate. Therefore, it is necessary to assess the performance of the tuning model with different length of recent ensemble activity. In addition, we also need to compare its performance with mov tuning model to demonstrate the superiority. Due to the point process property of a spike train, traditional distance measures like mean square error could not be applied directly. In this paper, we adopt the receiver operating characteristic (ROC) analysis and Kolmogorov-Smirnov (KS) plot to evaluate the performance of tuning models.

*Receiver Operating Characteristic Analysis.* Suppose the parameters of the tuning function have been estimated from the training data; then, for any neuron, we can compute the instantaneous firing rate *λ*(*t*). To get the ROC curve, we make a threshold and compute the spike prediction ${\hat{r}}_{c}(t)$ as follows:

(4) $$\begin{array}{c} {\hat{r}}_{c}\left(t\right)=1\;\text{if}\;\lambda \left(t\right)\geq c\\ {\hat{r}}_{c}\left(t\right)=0\;\text{if}\;\lambda \left(t\right)<c. \end{array}$$

For each threshold *c*, the ratio of the true positive rate (TPR) to the false positive rate (FPR) given the recorded spikes data could be computed, resulting in the ROC curve. The area under the curve (AUC) corresponds to the probability that the proposed model will assign a higher probability to the sample from the spike population compared to the sample from the no-spike population. Therefore it provides an assessment of the goodness of fit of the model.

*Kolmogorov-Smirnov Plot.* Based on the time-rescaling theorem, we can transform a point process into a Poisson process with unit rate which is appropriate for goodness of fit assessment [36]. Firstly, the tuning function is fitted to the spike train data based on the proposed model. Then the rescaled times *z*_*j*_ could be computed as follows:

(5) $$\begin{array}{c} z_{j}=1-\exp\left\{\overset{u_{j+1}}{\underset{u_{j}}{\int }}\lambda \left(t\right)dt\right\}, \end{array}$$

where *j* = 1,…, *J* − 1. If the proposed model is correct, *z*_*j*_ will be random variables sampled uniformly from the interval [0,1). The *z*_*j*_ values are ordered from smallest to largest, generating a new sequence *z*_*k*_. And a uniform density is defined as *b*_*k*_ = (*k* − 1/2)/*n* for *k* = 1,…, *n* against *z*_*k*_. Finally, the cumulative distribution function of the uniform density could be plotted, which is named KS plot. If the model is correct, points in the KS plot will be on a 45-degree line. More details can be found in [36].

### 3.2. Sequential Monte Carlo Estimation and Parallel Implementation

The state-space model based algorithms are widely used in brain machine interfaces to infer the latent state like the kinematics from neural activity. In this work, (3) and (2) constitute the observation model. In previous studies, the movement was often assumed to be a random-walk model, where the current state only depended on the previous state plus some noise. In this paper, we also incorporate the ensemble activity into the state vector, resulting in an augmented state vector *s*_*k*_ = [*x*_*k*_ *H*_*k*_], and then the new state equation is defined as

(6) $$\begin{array}{c} x_{k}=As_{k-1}+w, \end{array}$$

where *A* is the system evolution matrix and *w* is a zero-mean Gaussian noise with covariance matrix *Q*. As a result, the current state depends not only on the previous state, but also on the recent ensemble activity. We term the state-space model that includes the recent ensemble activity as the full model and whose state and observation models only depend on movements as mov model.

Once the transition and the observation functions have been defined as above, the state can be formulated as the posterior distribution given the observations, which can be estimated recursively as follows:

(7) $$\begin{array}{c} p\left(x_{k}\;|\;N_{1:k}\right)=\frac{p\left(x_{k}\;|\;N_{1:k-1}\right)p\left(dN_{k}\;|\;x_{k},H_{k}\right)}{p\left(dN_{k}\;|\;N_{1:k-1}\right)}, \end{array}$$

(8) p(xk ∣ N1:k−1)  =∫p(xk ∣ xk−1,Hk)p(xk−1 ∣ N1:k−1)dxk−1,

where *N*_1:*k*_ = [*dN*_1_, *dN*_2_,…, *dN*_*k*_] is the population activity up to time *k*, *p*(*x*_*k*_ | *N*_1:*k*−1_) is the one-step prediction which could be calculated according to (8), and the value of *p*(*dN*_*k*_ | *x*_*k*_, *H*_*k*_) can be computed based on the observation model.

An issue with the above recursive estimation is that (8) is difficult to compute because of the integration operation, and the posterior density is usually multimodes or highly skewed. Sequential Monte Carlo estimation provides a good solution to this problem [37]. The basic idea is to represent the posterior density as a set of weighted particles without restricting it to be any particular distribution. Initially, the weighted particles {*x*_1:*k*_^*i*^, *w*_*k*_^*i*^}_*i*=1_^*Ns*^ are generated from a prior density, and all the weights are set to 1/*Ns*. Then the algorithm runs in an iterative way. For each iteration, particles *x*_*k*_^*i*^ are generated from *p*(*x*_*k*_ | *x*_*k*−1_^*i*^, *H*_*k*_); then the importance weights are updated according to the equation *w*_*k*_^*i*^ = *w*_*k*−1_^*i*^ *p*(*dN*_*k*_ | *x*_*k*_^*i*^, *H*_*k*_). After the importance weights are all updated and normalized, the posterior density of state at time *k* can be approximated as

(9) $$\begin{array}{c} p\left(x_{k}\;|\;N_{1:k}\right)=\overset{Ns}{\underset{i=1}{\sum }}w_{k}^{i}k\left(x_{k}-x_{k}^{i},\delta \right), \end{array}$$

where *k*  (*x*_*k*_ − *x*_*k*_^*i*^, *δ*) is a Gaussian kernel whose mean is *x*_*k*_^*i*^ and covariance is *δ*. One principal purpose of BMI systems is to control the external device. A common way for the BMI decoder to output a control command is to average the posterior density. A phenomenon named degeneracy will appear after the algorithm runs over a few iterations, leading to a large amount of computational effort being wasted on the samples with small importance weights [38]. To overcome this problem, a resampling stage is introduced at the end of each iteration. In this paper, we adopt systematic resampling method and the details can be found in [39].

We adopt the root mean square error (RMSE) between the actual and the reconstructed trajectories as an assessment of the decoding algorithm and compare the decoding accuracy of the SMCPP based on the full model and mov model.

**Figure 2:**
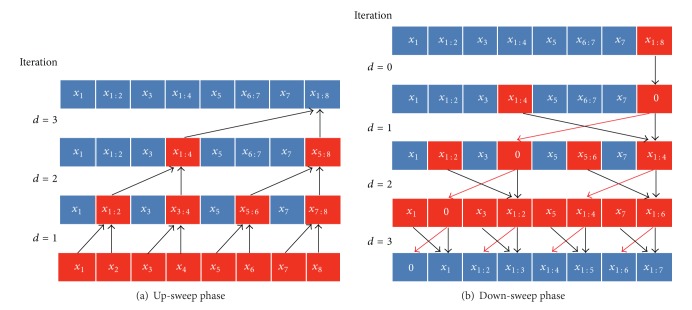
An illustration of the parallel prefix scan algorithm. (a) The up-sweep phase. (b) The down-sweep phase.

*Parallel Implementation.* To accelerate the computational speed, we implement the SMCPP in a massively parallel manner based on the compute unified device architecture (CUDA). It is computationally feasible for particles generation and weights calculation to concurrently execute, since the operation on each particle is independent of others. Then the resampling stage becomes a bottleneck because of its sequential in essence. In systematic resampling, the cumulation of the weights runs serially since the operation on current step is dependent on the result computed on last step. It takes *N* steps to get the result. We adopt parallel prefix sum algorithm which could dramatically reduce the computational complexity of calculating the cumulative sum [40]. The basic idea is to build a balanced binary tree based on the input data; then the prefix sum can be computed by sweeping the tree to and from its root. The algorithm is demonstrated in Figure 2, where the red blocks represent the active nodes and *x*_*i*:*j*_ = ∑_*k*=*i*_^*j*^ *x*_*k*_. The algorithm consists of two phases: the up-sweep phase which is depicted in Figure 2(a) and the down-sweep phase presented in Figure 2(b). During the up-sweep phase, at each iteration, half of the threads active at last iteration are still active and compute the partial sum of internal nodes with a distance of 2^*d*^. The down-sweep phase followed by the up-sweep phase is like the reverse of the previous phase. In each iteration, in addition to the computation of partial sum, each active node passes its values to its left child. The computational complexity of the parallel prefix sum algorithm is *O*(log⁡_2_ *N*), which is much more efficient than the serial version above whose computational complexity is *O*(*N*). More details of the algorithm can be found in [40].

## 4. Results and Analysis

A total number of 8 datasets recorded on different days are used in the following analysis. The summary of these datasets is listed in Table 1. Firstly, we determine the optimal length of the recent ensemble activity which enables the best performance of full tuning model. Then the performances of the mov tuning model and the full tuning model are compared. Since the importance weights are updated by the discrepancy between the neural firings and the tuning model, a well-defined tuning model is necessary to an accurate estimation of posterior density. We demonstrate the superiority of the tuning model considering the ensemble effect based on the receiver operating characteristic (ROC) analysis and Kolmogorov-Smirnov (KS) plot. For a high-performance real-time brain machine interface system, an accurate and fast prediction of the state is required. So we evaluate the decoding accuracy of the sequential Monte Carlo methods based on the mov model and the full model. Then, the decoding speed of the algorithm is compared between the parallel implementation running on GPU and the traditional one running on CPU.

### 4.1. Tuning Model Analysis

**Figure 3:**
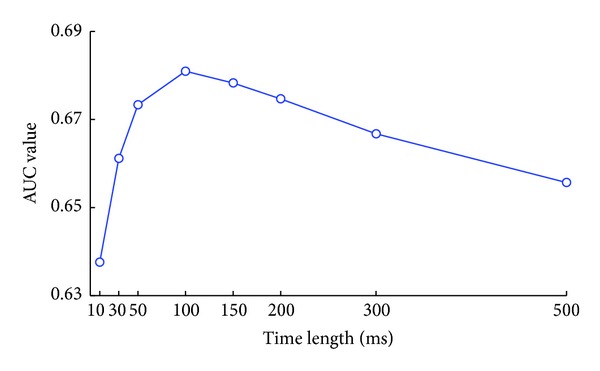
The AUC values of the full tuning model evaluated on all the neurons recorded. The* x*-axis represents the time length of recent ensemble activity included in the full tuning model.

We adopt the AUC values to assess the predictive performance of full tuning models with different length of recent ensemble activity. The AUC value, which is the area under the ROC curve, is dependent on all possible thresholds of the firing rate and computed by the true and false positive rates. If the model is perfect, the AUC value will be 1.  Figure 3 shows the relation between the time length of the ensemble activity and the AUC values which are averaged among all neurons recorded. We can observe that the AUC value increases rapidly as the more recent ensemble activity is included before 100 ms. However, when the time length exceeds 100 ms, the value decreases. It means that most information about the current firing probability is contained in the kinematics and the latest 100 ms ensemble activity. And incorporating the latest 100 ms ensemble activity with the kinematics could make the observation model gain the best performance.

**Figure 4:**
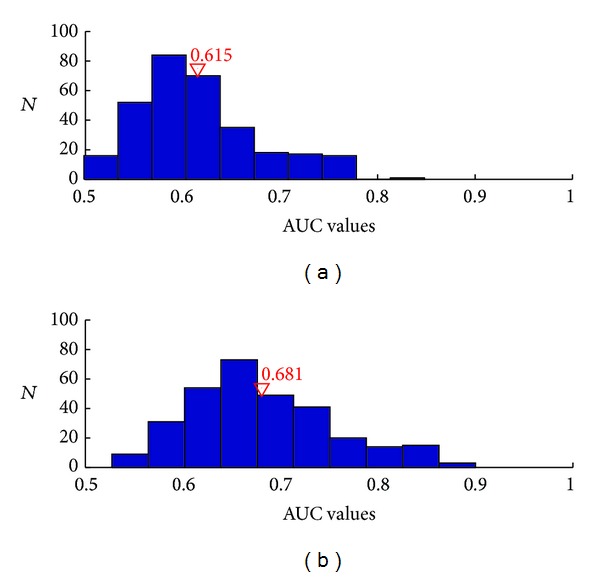
The histograms of AUC values of the tuning models evaluated on all the neurons recorded. The* x*-axis is the possible AUC values and* y*-axis is the number of neurons with the corresponding value. The numbers in red color are the averaged AUC values. (a) Mov tuning model. (b) Full tuning model with the recent 100 ms ensemble activity.

**Figure 5:**
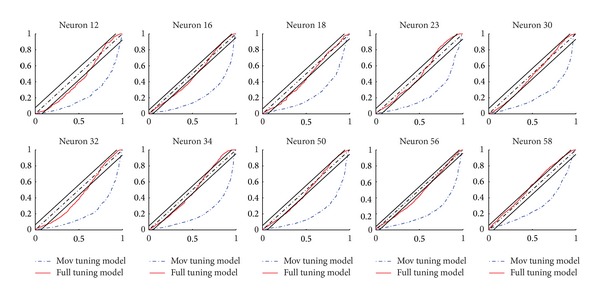
KS plots of 10 neurons for the mov tuning model and the full tuning model. The* x*-axis represents the quantiles and the* y*-axis represents the cumulative distribution function. The black thin lines in the figure represent the 95% confidence interval. Compared with mov tuning model, the improvement by full tuning model is considerable.

We also make a comparison of the predicting powers between mov tuning model and the full tuning model.  Figure 4 shows that histograms of AUC values evaluated on all the neurons in the total 8 datasets. The* x*-axis is the possible AUC values and* y*-axis represents the number of neurons with the corresponding value. The average AUC value for each model is shown in red color. Compared with the model only considering kinematics, the one including ensemble effect has a significantly better predictive performance.  Figure 5 shows the KS plots of 10 typical neurons. The* x*-axis represents the quantiles and the* y*-axis represents the cumulative distribution function with respect to the uniform distribution when the conditional intensity function equals the true one. It is easy to see that the mov tuning model tends to underestimate the conditional intensity especially at middle quantiles, while incorporation of the ensemble effect into the tuning model can greatly improve the explanation of the spike activity. The black thin lines in the figure represent the 95% confidence interval. We can observe that, for most neurons, the KS plots based on our proposed model fall within the 95% confidence limits. This improvement of the fitness demonstrates that, in addition to the kinematics, the recent ensemble activity affects the spiking of the current neuron.

### 4.2. Decoding Accuracy

**Figure 6:**
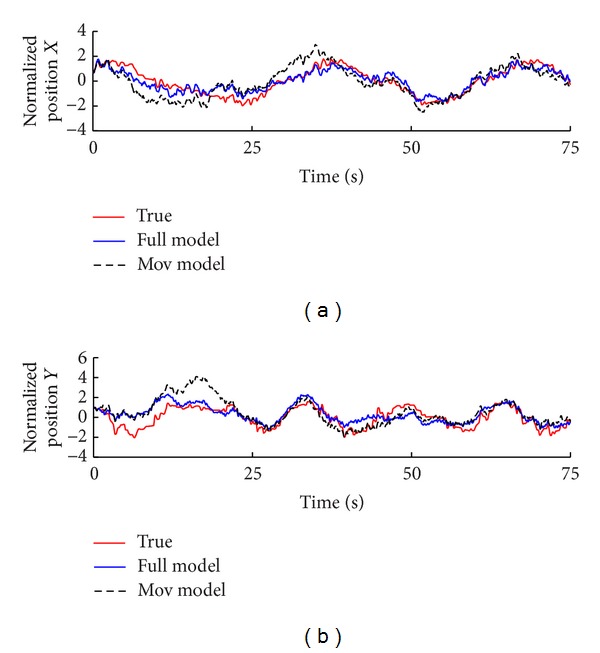
Reconstructed positions for a target-reaching task estimated by sequential Monte Carlo method. The red lines are actual movement, the blue lines represent the estimation based on full model, and the black dash lines are predicted based on the mov model.

Based on the models with and without the ensemble effect, we decode the neural activity recorded during the subject performing the movement task using sequential Monte Carl estimation.  Figure 6 shows a segment of the reconstructed kinematics. The upper and down panels, respectively, show the predicted kinematics for normalized position values on* x*-axis and* y*-axis. In each subplot, the red solid line indicates the true signal, the dash black line indicates the estimation by mov model, and the solid blue line is the estimation by the full model. It is obvious that the full model provides the more consistent reconstruction compared to the previous one.

**Figure 7:**
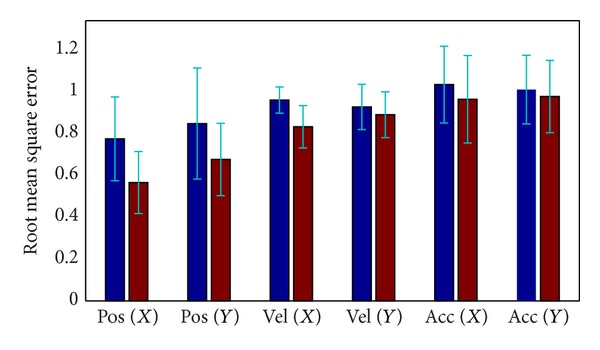
The statistical performance provided by the sequential Monte Carlo estimation with different models. Blue bars and red bars represent the mov model and full model, respectively. It is obvious that the full model can dramatically reduce the error during the estimation of movement trajectory.

We apply the two kinds of models on all of the 8 datasets to decode the kinematics. The first 200s of each dataset is used as training data, and the rest is for testing.  Figure 7 shows the statistical performance evaluated on the 8 datasets. The red and blue bars depict the decoding accuracy evaluated by the models with and without incorporating ensemble effect, respectively. It is clear that the decoding error is greatly reduced if the ensemble activity is combined with the kinematics in the model. Furthermore, among the kinematics the position gains the highest improvement (about 23.6%), and the improvement of the acceleration is not so much obvious (about 5.02%). Here we perform the left-tail paired Student* t*-test against the alternative that the decoding error of our proposed method is smaller. All the tests are performed on the null hypothesis at *α* = 0.05 significance level. The *P* values are shown in Table 2. Not surprisingly, the SMCPP method based on the full model provides smaller decoding error than the one based on mov model statistically (*P* < 0.05, left tail, paired Student's* t*-test). In most brain machine interface applications, we usually use the position values to control an external device. Therefore, the incorporation of ensemble effect in the decoding algorithm is helpful to high-performance BMI systems.

**Figure 8:**
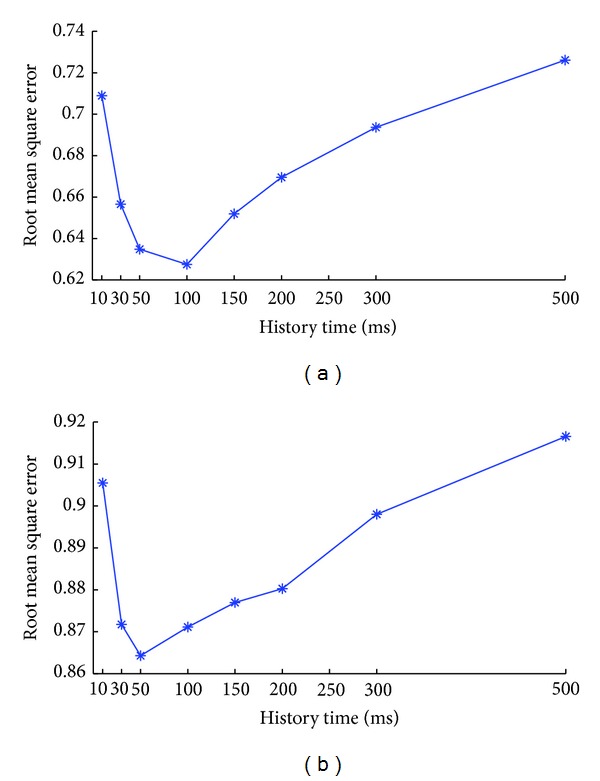
The decoding accuracy evaluated based on the tuning model with different time length of recent ensemble activity. (a) Average root mean square error of position. (b) Average root mean square error of velocity.

We vary the time length of the recent ensemble activity included in the full model and evaluate the corresponding decoding accuracy. The results are displayed in Figure 8. The* y*-axis of two subplots represents the root mean square errors evaluated on the position and velocity, respectively. The left subplot demonstrates that incorporating the latest 100 ms ensemble activity could achieve the best decoding result on position. It is consistent with the result obtained from Figure 3 that 100 ms is the best time length for the ensemble activity included in the tuning model. The right plot demonstrates that if we want the velocity to be accurately reconstructed, then the latest 50 ms ensemble activity is a better choice. However, regardless of the position or velocity, the corresponding decoding accuracy always improves at the beginning as more ensemble activity included and then drops when the time length continues to increase.

### 4.3. Decoding Speed

A high-performance real-time brain machine interface system requires not only the decoding result to be accurate but also the decoding speed to be fast enough. A challenge to the application of sequential Monte Carlo estimation in such kind of system is the high computational complexity. To accelerate the decoding speed, we implement the algorithm in fully parallel based on CUDA and compare the performance with the one that runs serially. The serial version of the algorithm runs on the i7 CPU with a clock rate of 2.4 GHz, and the RAM of the computer is 4 GB. The GPU used by our parallel algorithm is NVIDIA GT730M, in which the clock rate is 758 MHz, the number of CUDA cores is 384, and the global memory is 1 GB. The clock rate of CPU is much faster than the one of GPU.

**Figure 9:**
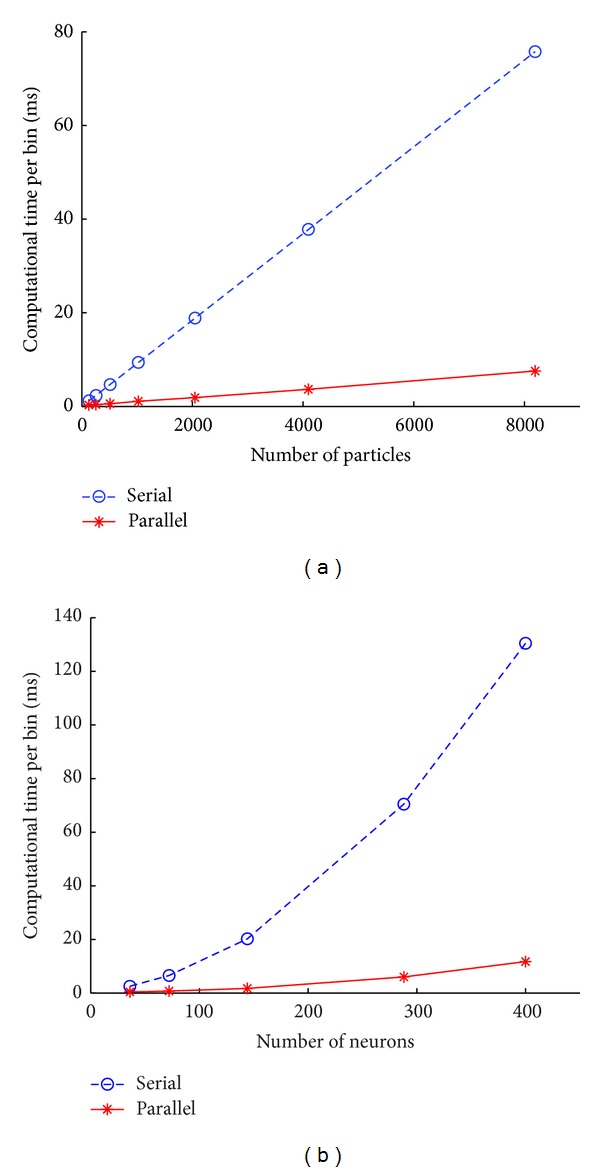
(a) The computational time of a single bin with different number of particles. (b) The computational time of a single bin with different number of neurons.

The experimental results are plotted in Figure 9. Figure 9(a) shows the relationship between the number of particles and the decoding speed of corresponding algorithms. The blue dash line represents the serial implementation running on CPU, and the red solid line is our proposed one running in parallel on GPU. As the number of particles increases, the computational time needed for each bin becomes much longer. For our parallel implementation, the computational time for each bin is less than 8 ms and even the number of particles is as large as 8000, while one for the serial version is as high as 80 ms. In neural decoding with point process observation, a common choice for the temporal resolution of spike trains is 10 ms, which means that our algorithm can work well in real-time brain machine interface applications even with a large number of particles. In addition, we evaluate the decoding time with different number of neurons. Since the number of neurons recorded is limited, we duplicate the neurons many times to get a large ensemble size. The results are plotted in Figure 9(b). It shows that when the number of neurons exceeds 100, for the serial version of the algorithm, the computational time of each bin is larger than 10 ms and increases rapidly as more neurons are used. While our proposed method can decode the neural activity in 10 ms even the number of neurons is as much as 300, which is feasible nowadays due to the development of single-unit recording technology.

## 5. Conclusion and Discussion

In this work, we attempt to improve the decoding performance of the SMCPP algorithm by addressing two issues in previous studies. One is the simplified tuning considering no neural ensemble effect, which may degenerate the decoding performance. The other is the high computational complexity, which brings the challenge for real-time implementation. We propose to include neural ensemble effect into the tuning function and find that 100 ms is the optimal time length of the ensemble activity which enables the model to perform the best, while previous SMCPP methods usually assume the instantaneous firings only depend on the movement. The goodness of fit analysis demonstrates that the tuning model which takes the ensemble effect into account can greatly increase the predicting power on the neuronal response. It is more consistent with the neurophysiologic knowledge since cortical neurons are interconnected in a large network by a huge number of synaptic inputs which can induce some kind of coupling [25].

Given the tuning function, the sequential Monte Carlo estimation can be applied directly on the spike trains to predict the movement. The posterior density of movement is represented by a set of weighted samples, and the importance weights are updated based on current neural firings and the instantaneous firing rate estimated from the tuning model. Therefore, the tuning model which is well consistent with the true one is necessary for a good estimation of states. In addition, we also incorporate the ensemble activity into the state transition equation. Based on the state and tuning models we propose, a SMCPP algorithm is built and applied to predict the movement from the neural activity recorded in primary motor cortex. We evaluate the statistical decoding accuracy on multiple datasets. The results demonstrate that our proposed model which takes the recent ensemble activity into account can improve the decoding accuracy, especially on position, compared to the model which only depends on the movement. Furthermore, we find that the best accuracy on position is achieved by including the latest 100 ms ensemble activity.

Though the SMCPP can predict the movement more accurately by taking the recent ensemble activity into account, the high computational complexity is still an issue which prevents it from being used in real-time brain machine interfaces. To accelerate the decoding speed, we implement the algorithm in fully parallel based on CUDA. The weighted particles are propagated, updated, and resampled simultaneously on hundreds of cores in GPU. The result shows, for our method, the computational time for each 10 ms data input is only about 8 ms and even the number of particles is larger than 8000. We also find that our algorithm can also fulfill the time resolution of real-time BMI systems and even over 3 hundred neurons are recorded. Compared with the serial implementation running on GPU, our parallel method runs over 10 times fast. Another advantage of our proposed algorithm is that the GPU is relatively cheap and simple to be upgraded compared with the CPU.

Our work enables the sequential Monte Carlo algorithm with point process observation to output the movement estimation much faster and more accurate, which is helpful to the high-performance BMI systems. Decoding accuracy can be improved by taking the recent ensemble activity into account. Meanwhile the decoding speed is much faster compared to the traditional ones running on CPU. Although the results are interesting, the signal processing approaches for spike trains can be further developed. A feasible way is to improve the encoding model by considering the sparseness of the neural connections. The more accurate model can reduce the bias during the update of the importance weights and is potential to increase the decoding accuracy.

## Figures and Tables

**Table 1:** The summary of the 8 datasets used in the analysis.

Datasets	1	2	3	4	5	6	7	8
Number of neurons	40	38	39	58	40	36	27	31
Signal-to-noise ratio	4.34	4.33	4.17	3.52	3.58	3.89	4.06	4.01
Firing rate (Hz)	8.15	10.50	9.90	6.66	9.83	6.72	5.81	4.31
Length (sec)	400	400	400	400	400	400	400	300

**Table 2:** *P* values (left-tail, paired Student's *t*-test, α = 0.05).

	Pos(*X*)	Pos(*Y*)	Vel(*X*)	Vel(*Y*)	Acc(*X*)	Acc(*Y*)
Full model versus mov model	2.58*e* − 5	0.0412	0.0053	0.0049	9.30*e* − 4	0.0372
